# Cognitive performance and engagement in physical, social and intellectual activities in older adults: The FIBRA study

**DOI:** 10.1590/1980-57642015DN93000010

**Published:** 2015

**Authors:** Giovana Sposito, Anita Liberalesso Neri, Mônica Sanches Yassuda

**Affiliations:** 1 Physiotherapist, doctoral student on the Post-graduate Program in Gerontology. School of Medical Sciences, State University of Campinas.; 2 Psychologist. Full Professor of the Post-graduate Program in Gerontology. School of Medical Sciences, State University of Campinas.; 3 Psychologist. Associate Professor of the School of Arts, Sciences and Humanities (EACH). University of São Paulo (USP) and Lecturer on the Post-graduate Program in Gerontology. School of Medical Sciences, State University of Campinas.

**Keywords:** activities of daily living, social participation, motor activity, cognition, elderly

## Abstract

Cognitive decline in aging can negatively impact quality of life in the elderly. However, studies have shown that elderly engaged in advanced activities of daily living (AADLs) can maintain or enhance global cognitive function or specific domains.

**Objective:**

To investigate the relationship between engagement in AADLs and domains of cognition in elderly from seven different locations in Brazil.

**Methods:**

A cross-sectional study involving 2,549 elderly without cognitive deficits suggestive of dementia was conducted. Data were collected on sociodemographic characteristics, health status, the Mini-Mental State Exam (MMSE) by subdomain (orientation, memory, attention/calculus, language and constructional praxis), and engagement in AADL grouped under physical, social and intellectual activities.

**Results:**

Multivariate linear regression analysis revealed an association, albeit modest, between intellectual AADLs and the domains orientation, attention/calculus, language and constructional praxis (R^2^=0.005, 0.008, 0.021, and 0.021 respectively). Social AADLs were correlated with memory (R^2^=0.002) and language (R^2^=0.004) domains. No association was found between physical AADLs and MMSE domains. Schooling and family income were the sociodemographic variables exhibiting the strongest relationship with cognitive domains.

**Conclusion:**

The study found associations between intellectual and social AADLs with higher cognitive performance, suggesting that active aging can provide opportunities to attenuate cognitive decline in aging.

## Abstract (pt)

O declínio cognitivo no envelhecimento pode interferir na qualidade de vida do idoso. No entanto, estudos tem documentado que idosos engajados em atividades avançadas de vida diária (AAVD) podem manter ou melhorar a função cognitiva global ou domínios específicos.

**Objetivo:**

Investigar a relação entre o engajamento em AAVD e domínios da cognição em idosos residentes em sete localidades brasileiras.

**Métodos:**

Participaram de estudo transversal 2.549 idosos sem déficits cognitivos sugestivos de demência. Foram coletados dados referentes às características sociodemográficas, de saúde, Mini Exame do Estado Mental (MEEM) subdividido em subdomínios (orientação, memória, atenção/ cálculo, linguagem e praxia construtiva) e o engajamento em AAVD agrupadas em físicas, sociais e intelectuais.

**Resultados:**

Embora modesta a análise de regressão linear multivariada mostrou associação entre as AAVD intelectuais e os domínios de orientação, atenção/ cálculo, linguagem e praxia construtiva (R^2^=0.005, 0.008, 0.021, 0.021 respectivamente). As AAVD sociais se associaram aos domínios de memória (R^2^=0.002) e linguagem (R^2^=0.004). Não houve associação entre a as AAVD físicas e os domínios do MEEM. Escolaridade e renda familiar foram as variáveis sociodemográficas que apresentaram relação mais robusta com os domínios cognitivos.

**Conclusão:**

O estudo mostrou associação entre as AAVD intelectuais e sociais e o melhor desempenho cognitivo, sugerindo que o envelhecimento ativo pode oferecer oportunidades para amenizar o declínio cognitivo no envelhecimento.

## INTRODUCTION

Among the morphophysiological and functional changes brought on by aging, cognitive performance is affected and some cognitive abilities decline.^1^ These changes are considered normal and generally do not affect global functioning.^1^ Changes are often seen in attention, information processing speed, performance of working and episodic memory, and in executive functions.^1,2^ However, individual differences, life-style and sociodemographic variables can lead to more marked cognitive decline^3,4^ and increase the risk of dementia which negatively impact autonomy, independence and quality of life of the elderly.^5,6^

In aging, it is necessary to differentiate normal from pathological cognitive decline, as early diagnosis of significant changes in cognitive performance allows better treatment, management and therapeutic planning for the elderly patient and their family members, as well as enabling interventions for prevention and for promotion of mental health in the elderly.^7,8^ Recent studies have explored the relationship between engagement in advanced activities of daily living (AADLs) and cognitive performance, as a means of favoring successful healthy aging.^9,10^ AADLs are part of the competencies related to daily functioning, but considered more complex as they depend on motivation and preservation of a group of physical and cognitive competencies enabling independent functioning and social participation in broader settings.^10-12^ Discontinuation of these activities can be an early marker of dependence for carrying out instrumental activities of daily living.^11-13^ Engagement in AADLs benefits health, autonomy, functioning and well-being of the elderly population.^14^ Such activities are known to play a protective role against cognitive decline and in the prevention and progress of dementia.^9^

Involvement in different types of complex activities favors and reflects good functioning of various cognitive domains. Intellectual and physical AADLs have been linked to superior results on tests of episodic memory, executive function and language.^15-17^ Engagement in social AADLs is associated with less decline in episodic, semantic and working memory, perception speed, visuospatial ability and global cognition in the aging process.^17^

Despite the fact that there is evidence of beneficial effects of performing AADLs on various cognitive functions, no systematic assessments investigating the influence of the frequency, intensity and duration of AADLs on cognition are currently available. In addition, there is a lack of consensus over which activities make up this construct, and also on their classification into specific domains.^9,19-21^ Information on which cognitive domains benefit most from the practice of AADLs by elderly remains scarce^15^ and also which modality or modalities (physical, social or intellectual) of AADL most influence cognition.

Therefore, the objective of the present study was to examine the relationship between engagement in physical, social and intellectual AADLs, and performance on the subdomains of the MMSE (orientation, memory, attention and calculus, language and praxis), controlling for the effect of sociodemographic (age, gender, schooling and family income) and health (number of diseases and depressive symptoms) variables in community-dwelling elderly.

## METHODS

This investigation drew on data from the FIBRA study (Study Network on Frailty in Brazilian Elderly – Unicamp). FIBRA is a multicenter, cross-sectional cohort study whose purpose is to investigate frailty profiles and relationships between this condition and sociodemographic, biological and psychosocial variables in urban community-dwelling elderly aged 65 years or older. The FIBRA study involved representative samples of elderly from seven different locations in Brazil. In each location, simple random sampling of urban census sectors was carried out where the sample size was calculated based on the ratio between the number of elderly envisaged and the universe of urban census sectors.^22^ Data were collected between June 2008 and September 2009.

The eligibility criteria used for recruitment, performed at households by trained personnel based on a pre-established script within each randomly selected census sector, were as follows: age 65 years or older, permanent residence at the domicile within the census sector, and no severe cognitive, communication, sensory or mobility deficits. The exclusion criteria adopted were:^23^

[a] problems affecting memory, attention, spatial or temporal orientation, and communication suggestive of dementia, or mention of this diagnosis reached by a physician, as reported by a family member;
[b] temporarily or permanently bedridden;
[c] stroke sequela, such as localized weakness or aphasia;
[d] Parkinson's Disease with severely impaired motricity, speech or affectivity;
[e] severe visual or auditory loss, hampering communication;
[f] terminal stage.

Data were collected during a single session of between 40 and 120 minutes conducted in a public, easily accessible place at an address, time and day arranged with the elder at the time of recruitment. During the session, measurements were taken for sociodemographic, anthropometric, clinical and frailty variables and dementia screening were carried out. The Mini-Mental State Exam (MMSE)^24^ was applied to detect cognitive impairment, using the mean cut-off scores for levels of schooling suggested by Brucki et al. (2003)^25^ minus one standard-deviation (17 for uneducated elderly; 22 for elderly with between 1 and 4 years of schooling; 24 for 5 to 8 years; and 26 for elderly with 9 or more years of schooling). Elderly scoring over the MMSE cut-off proceeded to additional self-report measures of physical health status, functioning, social engagement, depressive symptoms and life satisfaction. Only elderly who scored over the MMSE cut-off subsequently answered the questions on practice of AADLs, where these participants constituted the sample of the present analysis.

All participants signed a free and informed consent form for the study approved by the Research Ethics Committee of the School of Medical Sciences of Unicamp (report 208/2007, CAAE 39547014.0.10001.5404).

**Participants.** A total of 2,549 elderly with no cognitive deficits suggestive of dementia, selected from the universe of participants of the FIBRA Unicamp Study (n=3.478) based on the above-mentioned cognitive criteria took part in the present study. Of the 2,549 participants, 568 (22.28%) were from Belém (PA), 294 (11.53%) Parnaíba (PI), 239 (9.38%) Campina Grande (PB), 316 (12.40%) Poços de Caldas (MG), 673 (26.40%) Campinas (SP), 300 (11.77%) from the sub-district of Ermelino Matarazzo, in São Paulo (SP) and 159 (6.24%) from Ivoti (RS),

### Instruments and measures

*1. Mini-Mental State Exam (MMSE).^24,25^* This is a screening instrument for dementia in elderly comprising 30 questions. For this study, the MMSE was analyzed according to the domains: orientation (10 points), memory (6 points), attention/ calculus (5 points), language (8 points) and constructional praxis (1).^26^

*2. Sociodemographic variables.* Gender; age group (65-69 years, 70-74 years, 75-79 years and ≥ 80 years); years of schooling grouped under the classes never been to school, 1-4 years of schooling, 5-8 years and 9 years or more, and monthly family income in number of minimum salaries in the ranges <1.0; 1.1-3.0; 3.1-5.0; 5.1-10 and >10.

*3. Number of chronic diseases.* Data was obtained by applying a questionnaire with nine dichotic items on the existence of a medical diagnosis for the following conditions in the 12 months leading up to the interview: heart disease; stroke, infarction or ischemia; hypertension; diabetes; osteoporosis; arthritis or arthrosis/ lung diseases; cancer and depression. Affirmative answers were counted and categorized into the groups 0; 1-2; ≥3.

*4. Depressive symptoms.* Symptoms were assessed using the Geriatric Depression Scale (GDS),^27^ a depression screening instrument for the elderly containing 15 dichotomous items inquiring about emotional state in recent weeks. The cut-off score adopted for suspected presence of significant depressive symptoms was >6.

*5. Social AADL.* Practice of this type of AADLs was assessed by 8 closed-type questions with the possible answers. "never did", "stopped doing" and "still do", using a list devised from the literature.^11^ Activities included were: pay visits, receive visitors, frequent churches, temples or practice activities linked to religion, take part in meetings, parties and dances, participate in cultural events, drive a car, take day trips outside the city and undertake longer trips outside the city or country. The number of activities with the "still do" answer was calculated for each participant.

*6. Physical AADLs.* The questions were taken from the Brazilian version of the Minnesota Leisure Time Activity Questionnaire (MLTAQ)^28,29^ included in the FIBRA protocol. Physical AADLs encompassed practices involving physical activities, sport and house work, such as: light or vigorous walks; light or vigorous runs; use stairs by choice; cycling/ ballroom dancing; gymnastics in the home, gym or community center; hydrogymnastics; swimming in pool, lake or sea; weight training; volleyball; table tennis, and participants were also asked whether they engaged in any other physical exercise or sport not mentioned. Men were also asked whether they had played football or been a football referee. The following activities were also investigated: doing domestic chores. preparing food, cutting grass with electric or manual mower, removing weeds and keeping a previously established garden or vegetable patch, hoeing, turning the soil, composting, digging, planting or seeding to grow a garden or vegetable patch, making or repairing furniture or domestic utensils using tools, besides asking whether the individual performed any other domestic activities not listed. The activities practiced by each participant were summed.

*7. Intelectual AADLs.* The number of intellectual AADLs practiced was calculated based on the questions for leisure and relaxation activities taken from the Brazilian version of the Minnesota Leisure Time Activity Questionnaire (MLTAQ),^28,29^ such as: watching television, reading newspapers or magazines, playing table games, and practicing other leisure or relaxation activity not mentioned. Women were asked whether they did knitting, crochet, lacework, painting, handicrafts or collecting at home, and men were asked whether they did handicrafts, painting or collecting at home. The activities practiced by each participant were summed together.

**Statistical analysis.** Descriptive analyses of the continuous variables was performed by calculating means and standard deviations. The categorical variables were described as percentages and tested using the Chi-squared for different groups. The Mann-Whitney test was used to compare interval variables between the two groups. The Kruskal-Wallis test was used for comparison of values of the distributions of three or more groups.

Due to the sample size and the fact that the AADLs did not have a grading indicating the number of activities necessary to influence cognitive performance, the frequency distributions of the physical, social and intellectual AADLs were divided into quartiles (less active, not very active, very active and extremely active).^30^

On multivariate regression analysis (applying Stepwise Forward criteria), the scoring of the five domains of the MMSE was used as dependent variables while the remaining sociodemographic variables (gender, age, schooling, family income), health conditions (number of diseases and depressive symptoms), and AADLs (physical, social and intellectual) served as independent variables. The variables male gender, GDS<6 and lower quartile (less active) of the AADLs were used as a reference. The interval variables were rank transformed owing to the absence of a normal distribution.^31^

In order to avoid the multicollinearity often found among independent variables in regression analyses, the variance inflation factors (VIF) were analysed. In the present study, the VIF was found to lie between 1.01 and 1.77, well below 10, the cut-off point for potential multicolinearity.^32^ A level of significance of 5% (p<0.05) was adopted for all statistical tests.

## RESULTS

Of the 2,549 elderly that took part in the study, 65.71% were women and mean age was 72.32 (±5.55) years. Mean years of schooling was 4.37 (±3.99) and mean family income was 3.97 (±4.92) minimum wages (MW). The mean number of diseases reported was 2.02 (±1.33) and 79.54% had a score of < 6 on the GDS. Mean level of engagement in total AADLs (physical, social, intellectual) was 11.67 (±4.03) activities practiced. Performance on total MMSE and individual domain scores are given in Table 1 together with characteristics of the sample.

**Table 1:** Means and standard deviations for sociodemographic variables, number of diseases reported, depressive symptoms, AADLs and cognitive performance. FIBRA study, Unicamp, 2008-2009.

Characteristics	n	Mean (±SD)	Minimum	Maximum
Gender	2549			
Women (%)	1675 (65.71)			
Age	2549	72.32 (5.55)	65.00	96.00
Years of schooling	2547	4.37 (3.99)	0.00	27.00
Family Income (MW)	2202	3.97 (4.92)	0.00	72.00
Number of diseases	2549	2.02 (1.33)	0.00	7.00
GDS	2542	3.53 (2.68)	0.00	15.00
< 6 (%)	2022 (79.54)			
Total AADLs	2549	11.67 (4.03)	1.00	27.00
Physical AADLs	2549	3.94 (2.15)	0.00	14.00
Social AADLs	2549	5.53 (2.21)	0.00	12.00
Intellectual AADLs	2549	2.19 (1.04)	0,00	6.00
Total MMSE	2549	24.98 (3.08)	17.00	30.00
MMSE Orientation	2549	9.47 (0.86)	4.00	10.00
MMSE Memory	2549	4.84 (0.98)	0.00	6,00
MMSE Atten/Calc	2549	2.83 (1.74)	0.00	5.00
MMSE Language	2549	7.25 (0.96)	4.00	8.00
MMSE Praxis	2549	0.58 (0.49)	0.00	1.00

n: number of subjects. SD: standard deviation. MW: minimum wage. AADLs: advanced activities of daily living. MMSE: Mini-Mental State Exam. Atten/Cal: attention & calculus.

Table 2 depicts a comparison among the mean scores of the five domains of the MMSE according to sociodemographic variables, number of reported diseases and depressive symptoms. The means for the five domains of the MMSE were significantly higher in educated elderly. Participants with higher family income and absence of depressive symptoms (GDS<6) had higher mean scores for all domains except memory. There was no difference in the domain attention and calculus for age group.

**Table 2:** Comparison of mean scores by elderly on MMSE domains, according to sociodemographic variables, number of reported diseases and depressive symptoms (n=2,549). FIBRA Study, Unicamp, elderly, 2008-2009

		MMSE Orientation			MMSE Memory			MMSE Attention/Calc			MMSE Language			MMSE Praxis	
		Mean (±SD)	p-value		Mean (±SD)	p-value		Mean (±SD)	p-value		Mean (±SD)	p-value		Mean (±SD)	p-value
Gender			0.430			**<0.001**			**<0.001**			**<0.001**			**<0.001**
	Female	9.47 (0.85)			4.89 (0.95)			2.48 (1.75)			7.30 (0.94)			0.60 (0.49)	
	Male	9.48 (0.86)			4.74 (1.02)			3.49 (1.52)			7.15 (0.99)			0.53 (0.50)	
Age (years)			<0.001*			<0.001**			0.419			<0.001***			<0.001****
	65-69	9.58 (0.74)			5.01 (0.89)			2.88 (1.74)			7.34 (0.92)			0.61 (0.49)	
	70-74	9.50 (0.86)			4.84 (0.96)			2.85 (1.72)			7.23 (0.97)			0.61 (0.49)	
	75-79	9.34 (0.97)			4.71 (1.05)			2.78 (1.80)			7.19 (0.99)			0.51 (0.50)	
	≥80	9.28 (0.95)			4.54 (1.06)			2.70 (1.72)			7.11 (0.98)			0.49 (0.50)	
Years of schooling			**<0.001**^+^			**<0.001**^++^			**<0.001**^+++^			**<0.001**^+++^			**<0.001**^+++^
	0	8.80 (1.18)			4.62 (1.14)			1.36 (0.91)			6.08 (0.91)			0.21 (0.41)	
	1-4	9.58 (0.71)			4.83 (0.96)			2.79 (1.62)			7.39 (0.80)			0.60 (0.49)	
	5-8	9.67 (0.61)			4.96 (0.87)			3.51 (1.38)			7.69 (0.54)			0.70 (0.46)	
	≥9	9.81 (0.45)			5.05 (0.85)			4.29 (0.94)			7.88 (0.34)			0.87 (0.34)	
Family Income (MW)			**<0.001**^§^			0.537			**<0.001**^§§^			**<0.001**^§§§^			**<0.001**^§§§§^
	<1.0	9.17 (1.05)			4.77 (1.10)			2.14 (1.72)			6.93 (1.05)			0.47 (0.50)	
	1.1-3.0	9.37 (0.94)			4.82 (1.00)			2.47 (1.75)			7.05 }(1.02)			0.49 (0.50)	
	3.1-5.0	9.61 (0.71)			4.82 (0.91)			3.20 (1.93)			7.45 (0.85)			0.65 (0.48)	
	5.1-10.0	9.69 (0.64)			4.88 (0.96)			3.69 (1.42)			7.39 (0.63)			0.74 (0.44)	
	>10.0	9.77 (0.50)			4.95 (0.88)			4.26 (1.04)			7.77 (0.52)			0.86 (0.35)	
Number of diseases			0.722			0.309			**<0.001**^||^			**0.016**^||^			0.063
	0	9,52 (0.78)			4.77 (1.00)			3.14 (1.68)			7.16 (0.98)			0.54 (0.50)	
	1-2	9,48 (0.86)			4.87 (0.95)			2.87 (1.73)			7.29 (0.96)			0.60 (0.49)	
	3	9,45 (0.88)			4.82 (1.01)			2.64 (1.77)			7.22 (0.95)			0.55 (0.50)	
GDS			**<0.001**			0.053			**<0.001**			**<0.001**			**<0.001**
	< 6	9,51 (0.83)			4.86 (0.96)			2.96 (1.71)			7.31 (0.93)			0.60 (0.49)	
	≥ 6	9,33 (0.95)			4.76 (1.04)			2.31 (1.79)			7.03 (1.03)			0.50 (0.50)	

MMSE: Mini-Mental State Exam. Attention/Cal: Attention & calculus. SD: standard deviation. MW: minimum wage.

* 65-69≠75-79, ≥80; 70-74≠75-79, ≥80.

** 65-69≠70-74, 75-79, ≥80; 70-74 ≠ ≥80.

*** 65-69≠75-79, ≥80.

**** 65-69≠75-79, ≥80, 70-74≠≥80.

+ 0≠1-4, 5-8, ≥9, 1-4≠≥9, 5-8≠≥9.

++ 0≠1-4, 5-8, ≥9, 1-4≠ ≥9.

+++ 0≠1-4, 5-8, ≥9, 1-4≠5-8, ≥9; 5-8≠≥9.

§ <1.0≠3.1-5.0,5.1-10.0, >10.0; 1.1-3.0≠3.1-5.0, 5.1-10.0, >10.0.

§§ <1.0≠3.1-5.0,5.1 10.0, >10.0; 1.1-3.0≠3.1-5.0, 5.1-10,0, >10.0; 3.1-5.0≠5.1-10.0, >10.0; 5.1- 10.0≠>10.

§§ =<1.0≠3.1-5.0; 5.1-10.0, >10.0; 1.1-3.0≠3.1-5.0, 5.1-10.0, >10.0; 3.1-5.0≠>10.0.

§§§§ <1.0≠3.1-5.0, 5.1-10.0, >10.0; 1.1-3.0≠3.1-5.0, 5.1-10.0, >10.0; 3.1-5.0≠>10.0, 5.1 10.0≠>10.0;

| 0≠1-2,3;

|| 0≠1-2.

On the multivariate linear regression analysis, a significant association was observed between intellectual AADLs and the domains orientation, attention & calculus, language and praxis (with the exception of memory domain), whereas social AADLs were associated with memory and language domains. No association was found between physical activities and MMSE domains. Schooling was the variable most associated with the domains analyzed (Table 3).

**Table 3:** Multivariate linear regression analysis with sociodemographic variables, number of diseases reported, depressive symptoms and AADLs (social and intellectual) as independent variables and score on the 5 MMSE domains as dependent variables (n=2193). FIBRA network, Unicamp, 2008-2009.

Variables		Categories	Beta (SE)*	p–value	R2(partial)
MMSE Orientation					0.1165
	Schooling		0.21 (0.02)	**<0.001**	0.0987
	Age		–0.08 (0.02)	**<0.001**	0.0087
	Family Income		0.07 (0.02)	**0.002**	0.0046
	Intellectual AADLs	Not very active	107.65 (32.84)	**0.001**	
		Very active	88.96 (37.33)	**0.017**	
		Extremely Active	86.76 (49.98)	0.083	0.0045
MMSE Memory					0.0351
	Age		–0.13 (0.02)	**<0.001**	0.0226
	Schooling		0.07 (0.02)	**0.001**	0.0066
	Gender	Female	86.17 (30.60)	**0.005**	0.0036
	Social AADLs	Not very active	35.66 (36.22)	0.325	
		Very active	12.18 (47.19)	0.796	
		Extremely Active	96.77 (42.80)	**0.024**	0.0023
MMSE Attention/Calculus					0.3510
	Schooling		0.43 (0.02)	**<0.001**	0.2606
	Gender	Female	–405.05 (26.77)	**<0.001**	0.0745
	Family Income		0.09 (0.02)	**<0.001**	0.0062
	Intellectual AADLs	Not very active	88.10 (32.88)	**0.007**	
		Very active	180.75 (37.59)	**<0.001**	
		Extremely Active	102.5054)	**0.042**	0.0076
	Depressive Symptoms	≥6	–84.47 (31.94)	**0.008**	0.0021
MMSE Language					0.3816
	Schooling		0.47 (0.02)	**<0.001**	0.3445
	Gender	Female	127.41 (24.18)	**<0.001**	0.0085
	Intellectual AADLs	Not very active	213.42 (29.88)	**<0.001**	
		Very active	238.58 (34.49)	**<0.001**	
		Extremely Active	267.41 (46.72)	**<0.001**	0.0212
	Social AADLs	Not very active	64.70 (28.36)	**0.023**	
		Very active	42.63 (37.40)	0.254	
		Extremely Active	48.34 (34.90)	0.166	0.0037
	GDS	≥6	–60.61 (29.27)	**0.039**	0.0013
	Age		–0.03 (0.02)	**0.034**	0.0012
	Family Income		0.04 (0.02)	**0.037**	0.0012
MMSE Praxis					0.2082
	Schooling		0.29 (0.02)	**<0.001**	0.1800
	Intellectual AADLs	Not very active	128.37 (31.35)	**<0.001**	
		Very active	227.06 (35.76)	**<0.001**	
		Extremely Active	253.07 (48.15)	**<0.001**	0.0214
	Gender	Female	98.64 (25.48)	**<0.001**	0.0047
	Family Income		0.05 (0.02)	**0.015**	0.0021

Beta: estimated value or slope coefficient in regression line. SE: standard error of beta. p: level of significance. R2: coefficient of determination. MMSE: Mini-Mental State Exam. AADLs: advanced activities of daily living. GDS: Geriatric Depression Scale.

## DISCUSSION

The objective of the present study was to assess the relationship between engagement in AADLs (subdivided into physical, social and intellectual) and cognitive performance, as assessed on the five domains of the MMSE (orientation, memory, attention & calculus, language and praxis) in a sample of community-dwelling elderly, controlling for the effect of sociodemographic (age, gender, schooling and family income) and health (number of diseases and depressive symptoms) variables.

Besides benefits for physical health and psychological well-being,^33^ recent studies have shown that engagement in physical activity can play a key role in relation to cognitive decline and dementia.^8,34^ Wang et al.^15^ assessed engagement in AADLs and cognitive decline in 1,463 elderly without cognitive or physical impairment. The sample was followed for a mean of 2.4 years, and a significant relationship between engagement in physical AADLs and lower decline in episodic memory and language was noted. In a similar study, Barnes et al.^17^ analyzed the cognitive performance of a cohort of 349 healthy individuals aged 55 years or older in relation to the practice of physical activity over a five-year period. The authors found that individuals who practiced physical activities and attained a high level of cardiovascular fitness had better results on tests assessing executive functions, attention and global cognitive function compared to those with poorer cardiovascular fitness. However, no association was found in the present study between engagement in physical AADLs and the cognitive domains assessed by the MMSE. Possible explanation for this disparity may stem from the different methods used for assessing adherence to exercise programs (duration, intensity, frequency or metabolic consumption of the physical activities practiced) and the different cognitive domains analyzed.^9^ Moreover, in the present study, domestic activities were grouped under the domain of physical AADLs and these activities may not demand sufficient physical effort to attain metabolic spend or may not be performed consistently.

Engagement in social AADLs during aging tends to increase self-esteem and in theory can reduce social isolation and depression.^35^ A study by de Frias and Dixon^36^ examined the effect of engagement in AADLs (physical, social and intellectual) on cognitive performance among 570 older adults between 53 and 90 years of age followed for 4.5 years. The results showed that at base line those individuals engaged in social activities had better performance on tests of executive functions compared to individuals not engaging in these activities. James et al.^18^ examined the association of engagement in social activities with cognitive decline among 1,398 older adults without cognitive deficits at base line followed for up to 12 years through annual reassessments. Engagement in social activities was associated with less cognitive decline during follow-up. A one-point increase in social activity score was associated with a 47% reduction in the level of global cognitive decline. The rate of global decline was 70% lower in elderly frequently engaged in social activities compared to elderly with low frequency of this activity. A similar association was found in all five assessed domains (episodic memory, semantic memory, working memory, perception speed and visuospatial ability).

The cross-sectional cohort study of Krueger et al.. (2009)^37^ found an association between engagement of elderly in social activities and domains of memory, information processing speed and visuospatial abilities. The present study evidenced a significant, albeit modest, association between engagement in social AADLs and scores on the MMSE memory and language domains. This finding suggests that social activities might produce an enriched and stimulating environment which leads to greater engagement in complex social actions that can benefit cognitive performance.^8,38,39^ More complex social activities, such as engagement in political activities may involve greater cognitive demand to perform the task.^9^ Less complex social activities, such as paying family visits, may not be sufficiently stimulating to benefit cognitive performance in aging.^19-21^ Yet, exploring the complexity of social activities was beyond the scope of the present study.

The practice of intellectual AADLs was associated with scores on the domains of orientation, attention/calculus, language and praxis, but not with memory score. Although these associations were modest, recent studies have shown that intellectual AADLs are key for good cognitive performance in aging, and that such activities may have a protective role against cognitive decline and dementia progression.^9^ The longitudinal study of Wang et al.^15^ cited previously, showed that elderly engaged in intellectual activities had less decline on the domains of language, executive function and in global cognition. Wilson et al.^16^ analyzed engagement in intellectual activities of 801 older persons who were members of a catholic clergy and their association with lower risk of Alzheimer's disease (AD) during an average follow-up of 4.5 years. In a proportional hazards model controlled for age, gender and education, a one-point increase in engagement in intellectual activities was associated with a 33% lower risk of AD. Engagement in intellectual activities was also associated with less decline in global cognition (47%), working memory (60%) and information processing speed (30%). These results suggest that frequent participation in intellectual activities is associated with a lower risk of developing AD.

In the literature, the cognitive reserve model is the most widely accepted to explain the positive relationships between practice of AADLs and cognitive performance. According to the model, preservation of cognitive performance in aging takes places as a result of exposure to complex activities during the life cycle, such as education, complex occupational activities and engagement in activities (physical, social and intellectual).^38^ The concept of cognitive reserve suggests that previous experiences can influence neural processes and synaptic organization, rendering them more effective, adaptive and plastic, thereby delaying the onset of neural losses associated with aging.^38^ The cognitive reserve model has also been used to clarify the association between low schooling and greater cognitive decline, as well as the higher risk of developing dementia among low educated individuals.^38^

The results of the present study, and those of previous studies, could be interpreted differently, i.e. engagement in AADLs may also depend on the opportunities afforded by the environment and modulated by socioeconomic aspects. In other words, better opportunities in the environment result in healthier and more active elderly with superior cognitive functioning,^21,40^ therefore reverse causality cannot be ruled out.

The present study has several limitations. Although AADLs were grouped into categories, some activities were not purely social activities for example, some may include physical and mental components or vice a versa.^9,15^ Further studies investigating a clearer definition for this construct are warranted, along with descriptions of activities to be included in this context. Also, the frequency, intensity and duration of the AADLs practiced by the elderly were not measured, factors which may further influence cognitive performance.^9^ Additionally, the use of other cognitive instruments could allow these relationships to be explored in more depth in domains not addressed by the MMSE, such as executive functions and other memory subsystems. MMSE is an instrument for assessing global cognition, and exploring its domains is a limited resource and may have impacted the conclusion of the present study. Moreover, the use of the MMSE for cognitive screening may have allowed the inclusion of elderly with some degree of cognitive impairment.

In summary, the results of the present study suggest that active aging (based on social and intellectual activities) may be associated with cognitive performance. These findings can help guide public policies by ensuring that programs and services targeting the elderly promote engagement in such activities. 
